# Perceptions on and roadblocks to implementation of standardized nomenclature in radiation oncology: A survey from TG‐263U1

**DOI:** 10.1002/acm2.14359

**Published:** 2024-04-30

**Authors:** Elizabeth L. Covington, Krithika Suresh, Brian M. Anderson, Margaret Barker, Kathryn Dess, Jeremy G. Price, Alexander Moncion, Marissa J. Vaccarelli, Lakshmi Santanam, Ying Xiao, Charles Mayo

**Affiliations:** ^1^ Department of Radiation Oncology Michigan Medicine Ann Arbor Michigan USA; ^2^ Department of Radiation Oncology University of North Carolina at Chapel Hill Chapel Hill North Carolina USA; ^3^ Memorial Care Long Beach California USA; ^4^ Department of Radiation Oncology Fox Chase Cancer Center Philadelphia Pennsylvania USA; ^5^ Northwell Health Lake Success New York USA; ^6^ Medical Physics Department Memorial Sloan‐Kettering Cancer Center New York New York USA; ^7^ Department of Radiation Oncology University of Pennsylvania Philadelphia Pennsylvania USA

**Keywords:** nomenclature, standardization, survey, TG‐263

## Abstract

**Purpose:**

AAPM Task Group No. 263U1 (Update to Report No. 263 – Standardizing Nomenclatures in Radiation Oncology) disseminated a survey to receive feedback on utilization, gaps, and means to facilitate further adoption.

**Methods:**

The survey was created by TG‐263U1 members to solicit feedback from physicists, dosimetrists, and physicians working in radiation oncology. Questions on the adoption of the TG‐263 standard were coupled with demographic information, such as clinical role, place of primary employment (e.g., private hospital, academic center), and size of institution. The survey was emailed to all AAPM, AAMD, and ASTRO members.

**Results:**

The survey received 463 responses with 310 completed survey responses used for analysis, of whom most had the clinical role of medical physicist (73%) and the majority were from the United States (83%). There were 83% of respondents who indicated that they believe that having a nomenclature standard is important or very important and 61% had adopted all or portions of TG‐263 in their clinics. For those yet to adopt TG‐263, the staffing and implementation efforts were the main cause for delaying adoption. Fewer respondents had trouble adopting TG‐263 for organs at risk (29%) versus target (44%) nomenclature. Common themes in written feedback were lack of physician support and available resources, especially in vendor systems, to facilitate adoption.

**Conclusions:**

While there is strong support and belief in the benefit of standardized nomenclature, the widespread adoption of TG‐263 has been hindered by the effort needed by staff for implementation.  Feedback from the survey is being utilized to drive the focus of the update efforts and create tools to facilitate easier adoption of TG‐263.

## 1 INTRODUCTION

The report on standardizing nomenclature from Task Group 263 (TG‐263) of the American Association of Physicists in Medicine (AAPM) was a response to a great need for consistent terminology, improved communication, and infrastructure for process improvement and workflow management (including automation) within radiation oncology.^1^, ^2^, ^3^, ^4^, ^5^ The TG‐263 naming convention improved data quality and sharing at the clinic and national level, and was adopted by the Global Quality Assurance of Radiation Therapy Clinical Trials Harmonization Group (GHG) for the consensus guidelines of organ‐at‐risk delineation in clinical trials.^6^ TG‐263 has also been implemented in a collaborative quality initiative and data abstraction frameworks that collect and analyze practice patterns and patient outcomes.^7^, ^8^, ^9^, ^10^ However, there are still present challenges that prevent the complete adoption of the TG‐263 naming formalism.^11^

Several groups have published automated solutions for identifying, naming, or relabeling structures in accordance with the TG‐263 standard to improve compliance.^12^, ^13^, ^14^, ^15^ Another group performed a phased rollout with TG‐263 compliant templates that resulted in an increase from 68.2% to 97.8% of compliant structures over a span of 26 months.^11^ Although there are several solutions to the roadblocks associated with TG‐263 compliance, many challenges remain that may be unique, or shared, by individual clinics and would be valuable to understand prior to the release of the TG‐263 update (TG‐263U1).

The goal of this work, and the effort by the TG‐263U1 committee, was to anonymously survey physicists, dosimetrists, and physicians working in radiation oncology and request feedback on the challenges associated with the adoption of the TG‐263 standard. The survey was sent to all AAPM, AAMD, and ASTRO members. Member demographic information, basic utilization of TG‐263, adoption of advanced concepts of TG‐263, and treatment planning system vendors were queried. The results provided valuable information that the committee can use to address gaps and improve the adoption of the upcoming updated TG‐263 report.

## 2 METHODS

Survey questions were written during monthly TG‐263U1 committee meetings. Survey questions were chosen to evaluate a spectrum of concepts related to the adoption of TG‐263, such as the rate of adoption of TG‐263, the difference between adoption rates between targets and organs at risk, and roadblocks for implementation. Free‐text questions were added to allow for feedback from respondents on the perception of support of TG‐263 amongst different clinical roles and to share reasons for satisfaction or dissatisfaction with the standard.

Once the survey was created, it was directly emailed to all members of AAPM on February 3, 2022. The first reminder was sent on February 10, 2023, and a final reminder was emailed on February 26, 2022. The survey was sent to ASTRO members on February 9, 2022, in the ASTROgram newsletter. The survey was sent via email to AAMD members on February 10, 2022. The survey was also shared on social media via Twitter on the official AAPM and AAPM TG‐263U1 Twitter accounts on February 3, 2022. Reminders were shared on the TG‐263U1 Twitter account on February 18, 2023, and March 4, 2022. The survey was closed on March 10, 2022.

### 2.1 Statistical methods

Descriptive statistics (frequencies, proportions) are reported overall and by role, size (number of radiation therapy treatment units in the department), and type of institution. Comparisons of respondent characteristics and responses by role, size, and institution type were performed using chi‐squared or Fisher's exact tests. Institution type was defined as research or private/community, with respondents indicating that their institution type as “Other” or “Cancer Center” being classified into each group based on their specified institution. Statistical comparisons of paired responses (i.e., responses across multiple questions) were performed using a McNemar's test to compare across two questions or a conditional logistic Score test to compare across multiple questions. A mixed effects logistic model with a random intercept for respondents was used to make statistical comparisons between issues encountered while implementing TG‐263 among those that did not adopt TG‐263.

## 3 RESULTS

The survey had 463 responses with at least one question answered and 310 (77%) respondents completed the survey. Surveys were considered complete if the respondent submitted the survey, even if all questions were not answered. Due to the general distribution, we anticipate that the survey would have potentially reached individuals for whom it was not applicable. Thus, we assume that those who completed the survey represent our target population and present an analysis of responses for those with completed surveys. With a sample size of 310, the maximum margin of error at the 95% confidence level for the reported percentages is ±5.6%. The demographics of those with completed surveys are shown in Table 1. Table 2 shows the respondents’ utilization of a standardized nomenclature and level of adoption. The respondents’ perceived level of support for physicians, dosimetrists, and physicists is shown in Table 3. The use of advanced TG‐263 concepts, such as using a ∼ to indicate a partially contoured structure, is shown in Table 4.

**TABLE 1:** Demographics of survey respondents (*n* = 310 with completed surveys).

Location of respondents	
United States	258 (83%)
Canada	16 (5%)
Other	36 (12%)
Primary employment	
Private or Community Hospital	126 (41%)
Medical School or University Hospital	89 (29%)
Cancer Center	84 (27%)
Other	11 (3%)
Number of radiation therapy treatment units in the department	
1	44 (14%)
2	69 (23%)
3−5	104 (34%)
6+	87 (29%)
Did not answer	6
Clinical role	
Physicist	226 (73%)
Physician	12 (4%)
Dosimetrist	72 (23%)
Vendors used for contouring and/or treatment planning	
Eclipse	217 (70%)
Velocity	65 (21%)
Pinnacle	51 (16%)
MIM	119 (38%)
RayStation	67 (22%)
Other	74 (24%)

**TABLE 2:** Respondents’ utilization of standardized nomenclature (*n* = 310 with completed surveys).

Question	Response
Please rate your belief in the level of importance of a standard nomenclature in radiation oncology:	
Unimportant	4 (1%)
Slightly important	16 (5%)
Moderately important	34 (11%)
Important	114 (37%)
Very important	142 (46%)
Is your institution using TG‐263 nomenclature	
Yes, OARs only	48 (15%)
Yes, targets only.	10 (3%)
Yes, OAR and targets.	132 (43%)
No	120 (39%)
How often are you using TG‐263 nomenclature clinically	
Never	120 (39%)
Rarely	0 (0%)
Sometimes	20 (7%)
Often	85 (27%)
Always	85 (27%)
What level of adoption do you have of TG‐263 at your clinic?^a^	
Written policy/procedure to use standard.	67 (36%)
Verbal agreement	118 (64%)
What issues have you encountered while implementing TG‐263 nomenclature?^b^	
Lack of department policies	122 (39%)
Lack of software tools	65 (21%)
Misalignment with clinical practice	111 (36%)
Lack of clinical champion	110 (35%)
Lack of support from leadership	93 (30%)
Other:	62 (20%)
Is there another nomenclature standard in your clinic?^c^	
Yes	44 (37%)
No	76 (63%)
What is your current nomenclature standard?^d^	
“Standardizing Naming Conventions in Radiation Oncology”, IJROBP 84, 4, 2012	4 (9%)
In‐house standard	33 (75%)
Other:	7 (16%)
I have had trouble adopting TG‐263 for organs at risk:	
Yes	91 (29%)
No	219 (71%)
I have had trouble adopting TG‐263 for targets:	
Yes	136 (44%)
No	172 (56%)
Did not answer	2
If you are using a non‐TG‐263 standard and have no immediate plans to adopt TG‐263, please select why (select all that apply)^d^	
There is little perceived benefit to using TG‐263 beyond current standard	16 (36%)
The effort required to translate historical records from our current standard to TG‐263	9 (20%)
Staffing / implementation effort	19 (43%)
Other	18 (41%)
Do you plan on adopting TG‐263 nomenclature in the future?^c^	
Yes	39 (32.5%)
No	15 (12.5%)
Unsure	66 (55%)
Do you prefer to have laterality directly connected to the organ name?	
Yes (e.g., OpticNrv_R_PRV03 or R_OpticNrv_PRV03)	254 (82%)
No (e.g., OpticNrv_PRV03_R or R_PRV03_OpticNrv)	13 (4%)
No preference	43 (14%)

^a^ Asked of those at an institution that uses TG‐263 nomenclature, *n* = 190.

^b^ Percentages presented out of those with complete responses.

^c^ Asked of those at an institution that does not use TG‐263 nomenclature, *n* = 120.

^d^ Asked of those that indicated there was another nomenclature standard in their clinic, *n* = 44.

**TABLE 3:** Respondents indicate their perceived level of support by clinical role (*n* = 310 with completed surveys).

Question	Response
What is the level of physician support of TG‐263 nomenclature:	
Weak	121 (39%)
Moderate	137 (44%)
Strong	51 (17%)
Did not answer	1
What is the level of dosimetrist support of TG‐263 nomenclature:	
Weak	76 (25%)
Moderate	127 (41%)
Strong	106 (34%)
Did not answer	1
What is the level of physicist support of TG‐263 nomenclature:	
Weak	43 (14%)
Moderate	114 (37%)
Strong	153 (49%)

**TABLE 4:** Use of advanced concepts in TG‐263 (*n* = 310 with completed surveys).

Question	Response
Do you use “!” to indicate when a high dose target volume has been removed from a low dose target volume (e.g., PTV!_Low)?	
Yes	22 (7%)
No	276 (89%)
Unsure	11 (4%)
Did not answer	1
Do you use “∼” to indicate a partially contoured organ at risk (e.g., Liver∼)	
Yes	18 (6%)
No	280 (90%)
Unsure	12 (4%)
Is your clinic using the TG‐263 nomenclature for dose‐volume histogram metrics (e.g., V20Gy[%] < 33)?	
Yes	167 (54%)
No	100 (32%)
Unsure	43 (14%)

**FIGURE 1:**
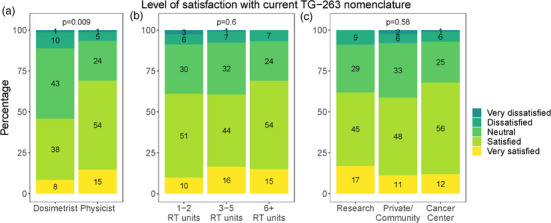
Level of satisfaction with the current TG‐263 nomenclature by role, size of clinic (number of RT units), and type of institution.

While this survey was sent via email to members of AAPM, AAMD, and ASTRO, physicists were the majority of respondents with over 70% identifying as a physicist in their clinical role, as shown in Table 1. Part of this difference can be attributed to the benefit of utilizing multiple reminders to AAPM members throughout the duration of the survey while ASTRO and AAMD had only one mass email solicitation and reliance on social media for additional reminders. The response rate can also be attributed to the trends shown in the perception of support by clinical role where physicists were perceived as having the highest level of support, as shown in Table 3. While the majority of respondents indicated their belief that a standardized nomenclature is important in radiation oncology, as shown in Table 2, the proportion of those who perceived strong support of TG‐263 (vs. moderate or weak) by physicists (49%), dosimetrist (34%), and physicians (17%) was significantly different (*p* < 0.001), as shown in Table 3. Physicists were statistically more likely to indicate a higher level of satisfaction (*p* = 0.009) when compared with dosimetrists, as shown in Figure 1(a). The level of satisfaction was not statistically significantly different when comparing the size or type of institution, as shown in Figure 1(b, c).

**FIGURE 2:**
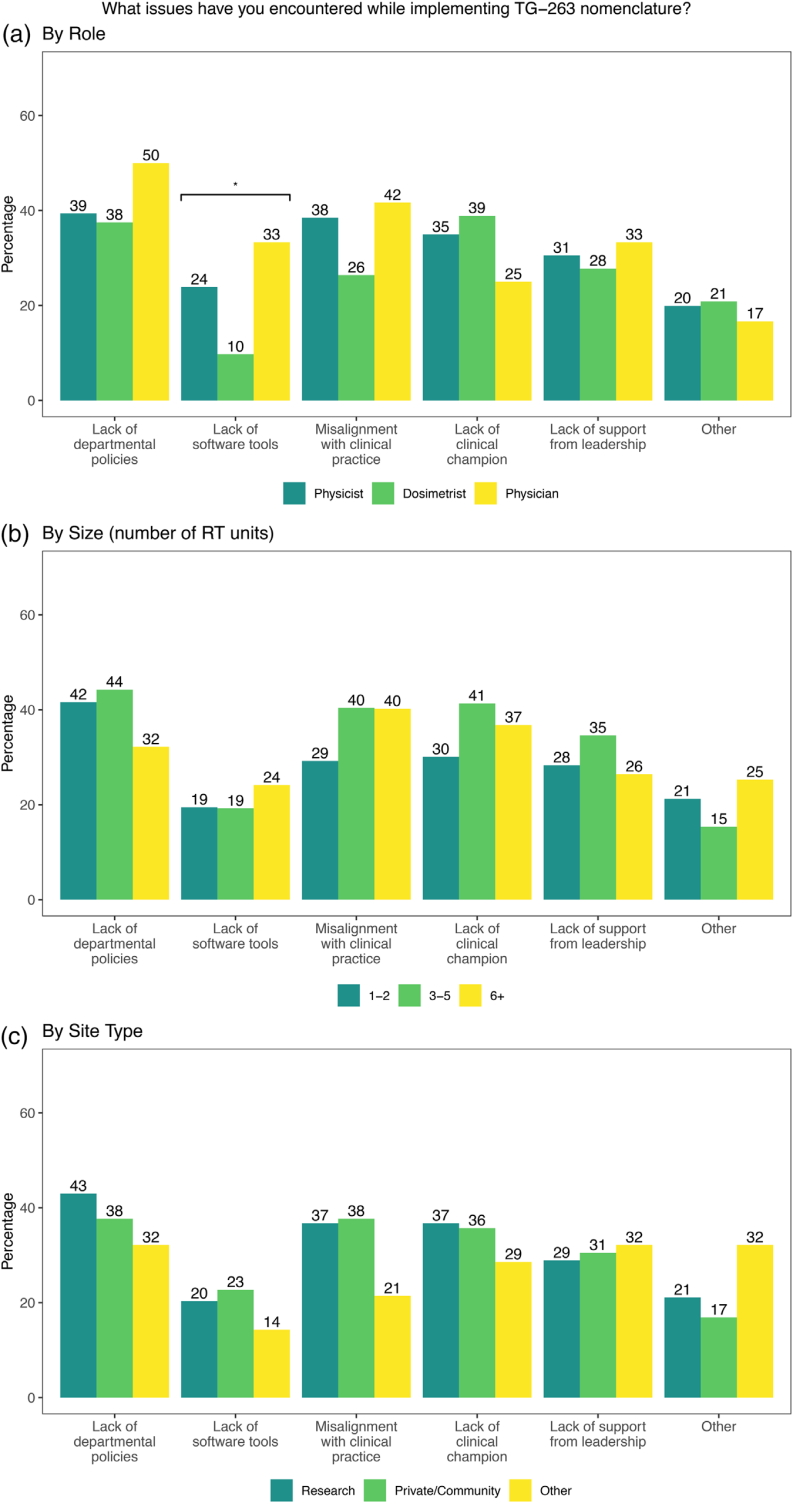
Responses to “What issues have you encountered while implementing TG‐263 nomenclature” by (a) role (b) size of clinic (number of RT units) and (c) by type of institution. Statistically significant differences are indicated for *p* < 0.05 (*).

Respondents indicated more difficulty in adopting TG‐263 for targets (44%) rather than OARs (29%), which was shown to be statistically significant (*p* < 0.001), as shown in Table 1. In free‐text responses, many respondents attributed this to a lack of physician support or adherence to using TG‐263 by physicians when naming targets. One respondent noted that “OARs are fairly straightforward and useful because we can build them into templates and have better control over the dosimetry policies than physician policies.” This matches themes in the free text comments about physician support being a barrier to both adoption and adherence to the standard and notable quotes are highlighted in Table 5. When asked what issues were encountered when adopting TG‐263, the only statistically significant difference between roles, clinic size, and type of institution was that physicists were more likely to indicate a lack of software tools as shown in Figure 2. For those who indicated that they had not adopted TG‐263 (*n* = 120), the odds of identifying a lack of software tools as an issue were significantly decreased compared to other issues such as leadership support (*p* = 0.001) and lack of clinical champions (*p* = 0.007).

**TABLE 5:** Common themes in free‐text responses on barriers to TG‐263 implementation.

Theme	Notable Quotes
Lack of vendor tools	“Any effort to adopt naming standards for treatment planning should be pushed via the vendor of the TPS, and not direct implementation at the clinic level.” “Vendor character limits make some of the rules create unrecognizable names.” “Vendors don't provide enough tools to help make standards more “automatic”.” “It would be great if it could be implemented as a standard by all vendors, so local staff did not have to spend the time setting it up, holding meetings to discuss the changes, having to come up with compromises to meet the needs of the system wide team.”
Lack of physician support	“Physicians are usually supportive in principle, but not always when it comes to the specific implementation…” “Physicians do not want to change; dosimetrist does not want to fight physicians…” “MD consistency in adopting target naming has been the biggest struggle.” “It has been a challenge because the physicians are resistant to change.”

**FIGURE 3:**
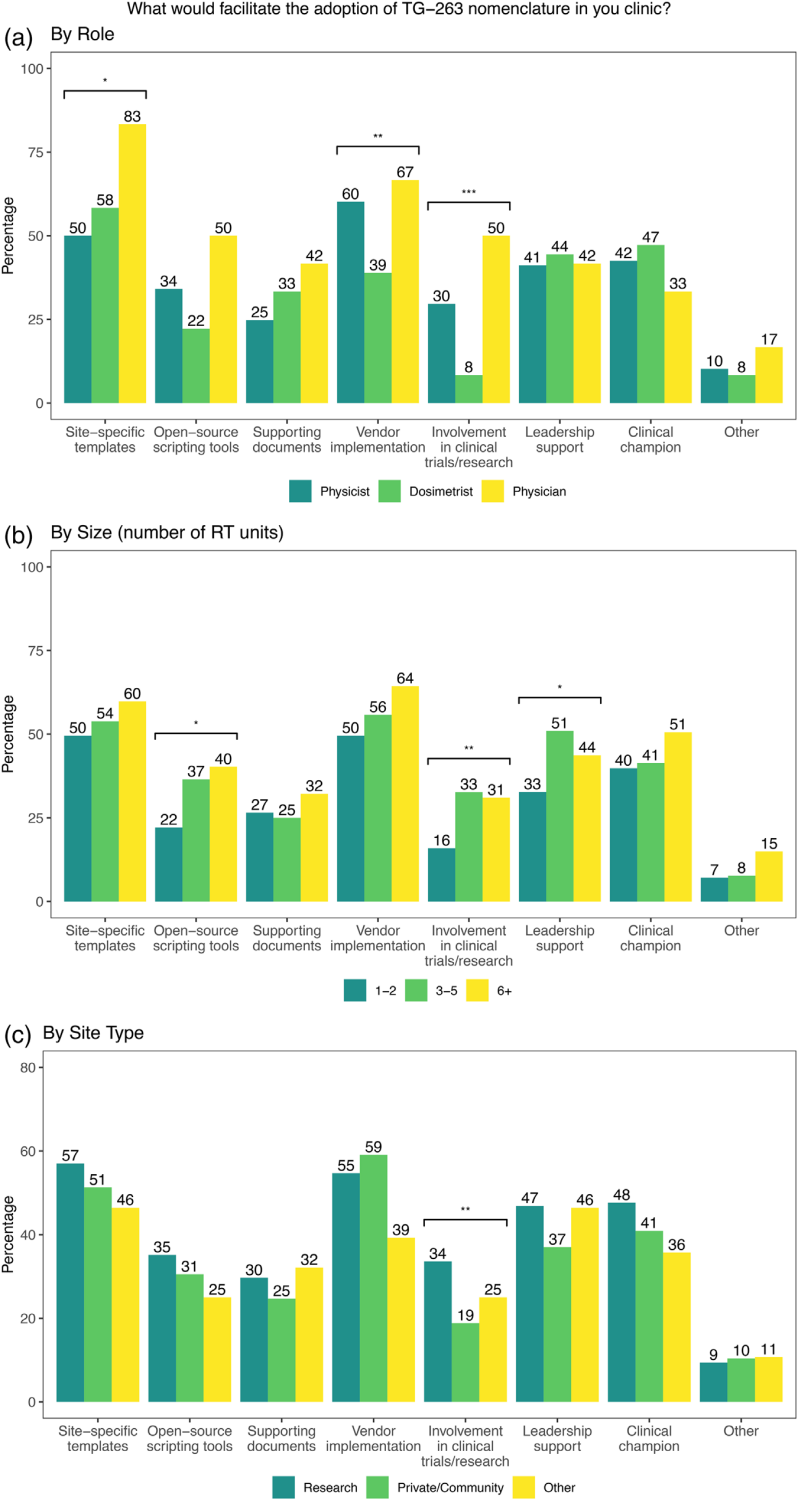
Responses to “What would facilitate the adoption of TG‐263 nomenclature in your clinic?” by (a) role (b) size of clinic (number of RT units) and (c) by type of institution. Statistically significant differences are indicated for *p* < 0.05 (*), *p* < 0.01 (**), *p* < 0.001 (***).

Figure 3 shows responses on what would facilitate the adoption of TG‐263 nomenclature, and there were statistically significant differences between physicists and dosimetrists on what would facilitate the adoption of the standard. Physicists and physicians were more likely to indicate that vendor implementation (*p* = 0.002) and involvement in clinical trials (*p* < 0.001) would facilitate adoption, as shown in Figure 3(a). Respondents at research institutions were more likely than those at community or practice institutions to indicate that adoption would be facilitated by leadership support (47% vs. 37%) as well as participation in clinical trials (34% vs. 19%), as shown in Figure 3(c). For respondents at clinics that have adopted TG‐263 (*n* = 190), 58% of those at research institutions are utilizing scripting compared to 38% at community or private clinics.

**FIGURE 4:**
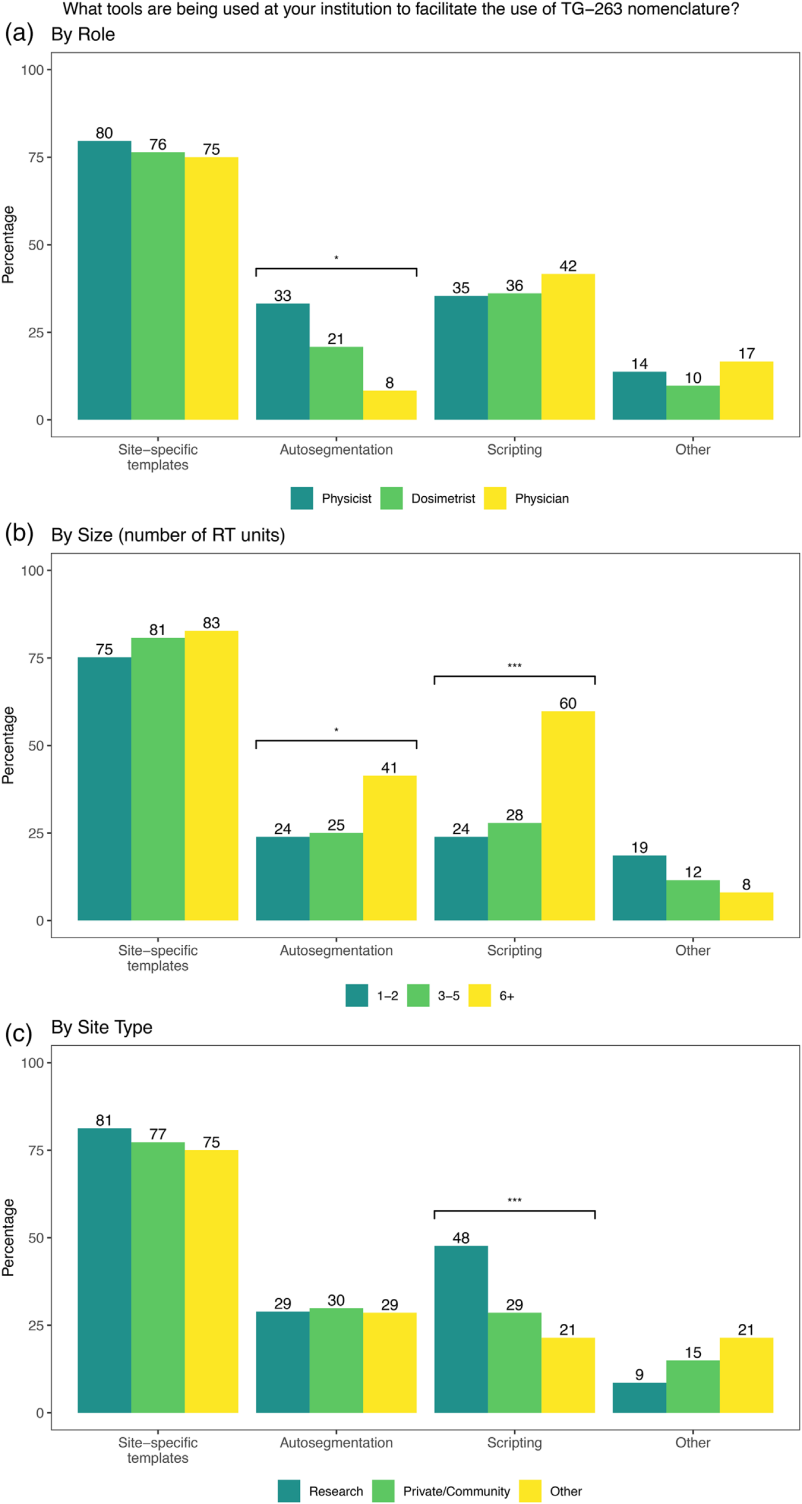
Responses to “What tools are being used at your institution to facilitate the use of TG‐263” by (a) role (b) size of clinic (number of RT units) and (c) by type of institution. Statistically significant differences are indicated for *p* < 0.05 (*), *p* < 0.01 (**), *p* < 0.001 (***).

Overall, vendor implementation (60%) and site‐specific templates (50%) were the two highest resources listed by physicists that would facilitate TG‐263 adoption, as shown in Figure 3. Notable quotes on vendor implementation are shown in Table 5. Physicians were more likely to indicate site‐specific templates (83%) as facilitating adoption, as shown in Figure 3(a). Site‐specific templates were indicated by all roles to be the most utilized tool for facilitation use of TG‐263, as shown in Figure 4(a). Institutions with six or greater treatment units were more likely to indicate that autosegmentation (*p* = 0.013) and scripting (*p* < 0.001) were being utilized, and research institutions were more likely to indicate scripting, as shown in Figure 4(b, c).

**FIGURE 5:**
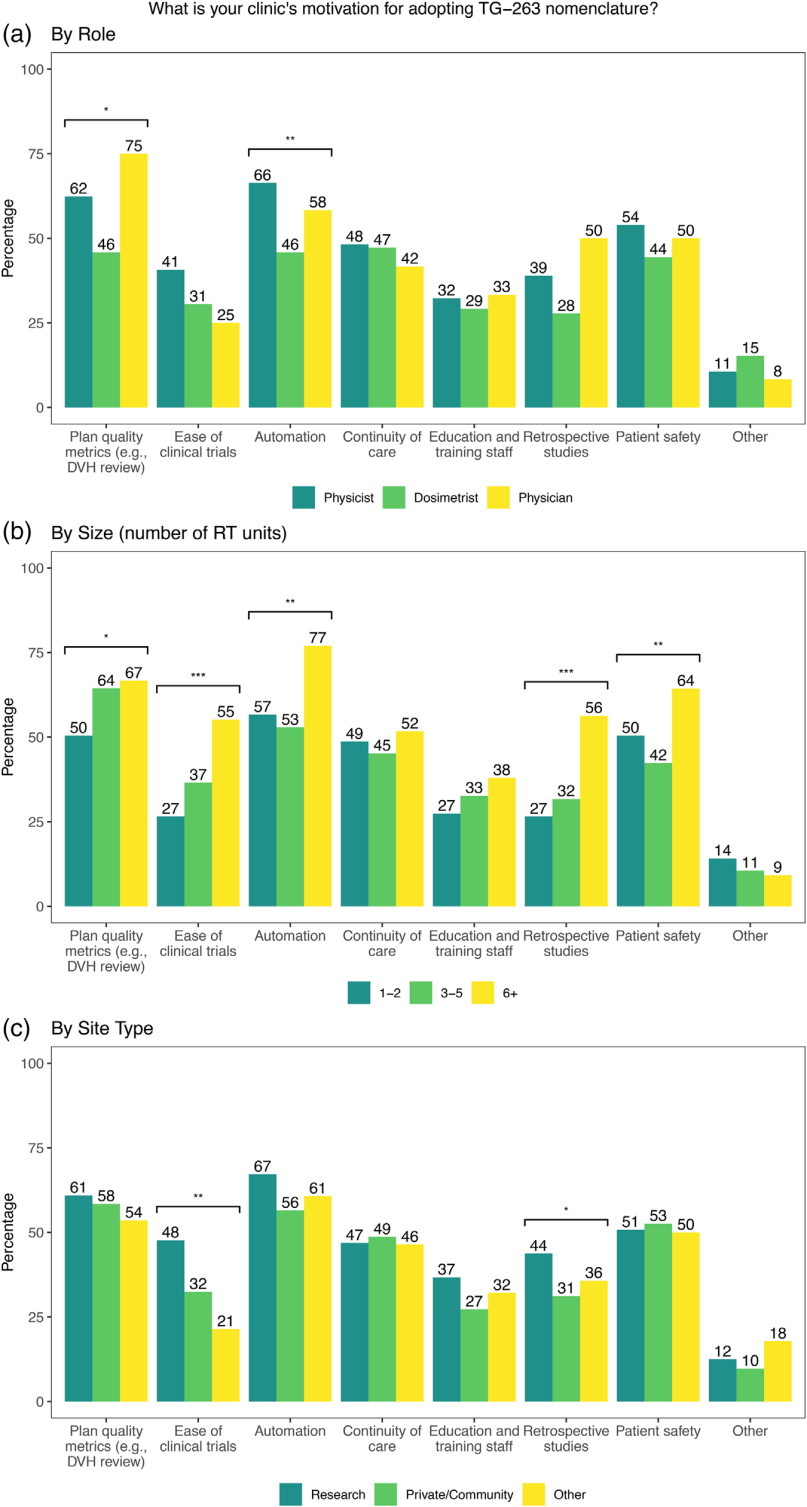
Responses to “What is your clinic's motivation for adopting TG‐263 nomenclature?” by (a) role (b) size of the clinic (number of RT units) and (c) by type of institution. Statistically significant differences are indicated for *p* < 0.05 (*), *p* < 0.01 (**), *p* < 0.001 (***).

There were also significant differences in the motivation for utilizing standardized nomenclature as shown in Figure 5. Physicists were more likely to indicate automation (*p* = 0.002) and evaluation of plan quality metrics (*p* = 0.013) as motivation when compared to dosimetrists, as shown in Figure 5(a). Continuity of care was selected by dosimetry most often as a motivator but was not statistically different from physicists. Motivation also varied by the size of the institution with those at sites with six or more treatment machines more often indicating ease of clinical trials (*p* < 0.001), automation (*p* = 0.001), retrospective studies (*p* < 0.001), and patient safety (*p* = 0.009) as motivation for adoption, as shown in Figure 5(b). Quotes with common themes on patient safety and automation are shown in Table 6. Similarly, those at academic institutions were more likely to indicate ease of clinical trials (*p* = 0.009) and retrospective studies (*p* = 0.029) as motivation, as shown in Figure 5(c).

**TABLE 6:** Common themes on benefits of adopting TG‐263.

Theme	Notable Quotes
Automation	“Physicists develop the scripts or automation tools so see a strong benefit of adopting the standardized nomenclature.” “Great effort by the AAPM that we have found very useful for facilitating automated plan review.” “Very useful for standardization and automation, need more guidelines like this.”
Safety and Quality	“… our therapists have adapted and easily know which volumes to have on for imaging as it is consistent from patient to patient.” “We adopted an implemented TG‐263 a year ago or so and it has made plan evaluation more consistent between dosimetrists, physicists and radiation oncologists.” “Clarity in patient chart, e.g. when sharing plan information with other centers of doing retrospective reviews.”

## 4 DISCUSSION

A motivating factor for TG‐263 was using a standardized nomenclature to add value to radiation oncology by providing a basis for improved, more consistent communication and the ability to develop automated solutions for data extraction and quality assurance to improve clinical workflows, safety, and research. The lack of functionality in commercial systems to incorporate needed metadata about the structures and dose metrics left the character string for the name as the only common element for consistently conveying needed information through a standardized nomenclature. Developing the standardization through multi‐institutional, multi‐professional society‐based consensus was and continues to be used to provide objective guidelines to both clinics and manufacturers.

Adoption has been strongest among institutions participating in clinical trials or developing and/or using script‐based automation where consistency is needed to reduce manual efforts to collect and use data supporting research practice. To lower the barrier to creating and maintaining site‐specific templates, an effort led by TG‐263U1 members has created an open‐source tool to create importable TG‐263‐compliant site‐specific templates.^16^ The single most helpful step would be vendors utilizing the professional society‐based standardized nomenclature and creation of templates to streamline clinical practice. Without that, the extra effort and resources depend strongly on clinical champions and support from leadership.

Among clinics that have adopted the standardization, a strong motivating factor is the ability to automate gathering and using data to inform practice quality improvement. Consistency in nomenclature enables accuracy in learning from historical data on how to improve care for future patients. Due to increasing requirements on data sharing for both funding agencies and scientific journals, required use of the standard by scientific journals in radiation oncology would further motivate adoption for academic centers. For both academic and community practices, recommendation from accreditation programs would also motivate adoption. Vendors adding functionality to electronic health records systems, radiation oncology information systems, and treatment planning systems so that end users can learn from their data is highly desirable to support practice improvement and would naturally lead to more consistent use of standards.

## 5 CONCLUSIONS

While there is consensus on the importance of having a standardized nomenclature in radiation oncology, respondents indicate barriers to implementation. The information shared in this survey is being used to guide the updates in TG‐236U1, including the creation of open‐source software tools and addressing perceived gaps in the standard.

## AUTHOR CONTRIBUTIONS

Elizabeth L. Covington, Brian M. Anderson, Margaret Barker, Kathryn Dess, Jeremy Price, Alexander Moncion, Marissa Vaccarelli, Lakshmi Santanam, Ying Xiao, Charles Mayo made substantial contributions to the conception or design of the work as part of Task Group TG‐263U1. Krithika Suresh, Elizabeth L. Covington, and Chuck Mayo made substantial contributions to the analysis and interpretation of data. All authors participated in drafting the work or revising it critically for important intellectual content. All authors had final approval of the manuscript and agree to be accountable for all aspects of the work in ensuring that questions related to the accuracy or integrity of any part of the work are appropriately investigated and resolved.

## CONFLICT OF INTEREST STATEMENT

Elizabeth L. Covington, Brian M. Anderson, Margaret Barker, Kathryn Dess, Jeremy Price, Alexander Moncion, Marissa Vaccarelli, Lakshmi Santanam, Ying Xiao, Charles Mayo are members of AAPM Task Group TG‐263U1.
